# Hexokinase 3 dysfunction promotes tumorigenesis and immune escape by upregulating monocyte/macrophage infiltration into the clear cell renal cell carcinoma microenvironment

**DOI:** 10.7150/ijbs.58295

**Published:** 2021-06-01

**Authors:** Wenhao Xu, Wang-Rui Liu, Yue Xu, Xi Tian, Aihetaimujiang Anwaier, Jia-Qi Su, Wen-Kai Zhu, Guo-Hai Shi, Gao-Meng Wei, Yong-Ping Huang, Yuan-Yuan Qu, Hai-Liang Zhang, Ding-Wei Ye

**Affiliations:** 1 Department of Urology, Fudan University Shanghai Cancer Center, Shanghai Medical College, Fudan University, Shanghai, 200032, P.R. China.; 2 Department of Urology, Affiliated Hospital of Youjiang Medical University for Nationalities, Baise, 533000, P.R. China.; 3 Department of Ophthalmology, Dushuhu Public Hospital Affiliated to Soochow University, Suzhou, 215000, P.R. China.

**Keywords:** clear cell renal cell carcinoma, HK3, immune checkpoint therapy (ICT), glycolysis, tumor microenvironment

## Abstract

**Purpose:** This study aimed to identify the potential prognostic role of HK3 and provide clues about glycolysis and the microenvironmental characteristics of ccRCC.

**Methods:** Based on the Cancer Genome Atlas (TCGA, n = 533) and Gene expression omnibus (GEO) (n = 127) databases, real-world (n = 377) ccRCC cohorts, and approximately 15,000 cancer samples, the prognostic value and immune implications of HK3 were identified. The functional effects of *HK3* in ccRCC were analyzed *in silico* and *in vitro*.

**Results:** The large-scale findings suggested a significantly higher *HK3* expression in ccRCC tissues and the predictive efficacy of *HK3* for tumor progression and a poor prognosis. Next, the subgroup survival and Cox regression analyses showed that *HK3* serves as a promising and independent predictive marker for the prognosis and survival of patients with ccRCC from bioinformatic databases and real-world cohorts. Subsequently, we found that *HK3* could be used to modulate glycolysis and the malignant behaviors of ccRCC cells. The comprehensive results suggested that *HK3* is highly correlated with the abundance of immune cells, and specifically stimulates the infiltration of monocytes/macrophages presenting surface markers, regulates the immune checkpoint molecules PD-1 and CTLA-4 of exhaustive T cells, restrains the immune escape of tumor cells, and prompts the immune-rejection microenvironment of ccRCC.

**Conclusion:** In conclusion, the large-scale data first revealed that *HK3* could affect glycolysis, promote malignant biologic processes, and predict the aggressive progression of ccRCC. *HK3* may stimulate the abundance of infiltrating monocytes/macrophages presenting surface markers and regulate the key molecular subgroups of immune checkpoint molecules of exhaustive T cells, thus inducing the microenvironmental characteristics of active anti-tumor immune responses.

## Introduction

Renal cell carcinoma (RCC) has become one of the most common malignancy of the genitourinary system 1, accounting for about 5% of all new adult male cases and 3% of all new female cases 2. In 2019, there were about 73,820 new cases of kidney cancer and 14,770 deaths in the United States. In China, there are about 66,800 new cases of kidney cancer each year, ranking second in the incidence of urinary system tumors. Clear cell renal cell carcinoma (ccRCC) is the most common and a highly malignant pathological type of RCC, accounting for 70% - 85% of all patients. About 25% - 30% of patients with ccRCC are first diagnosed when metastasis occurs, and the 5-year survival rate of patients with advanced ccRCC is merely 32%. For clinically limited tumors, the treatment is still based on nephron sparing surgery or radical nephrectomy intervention. Further cytokine or individualized precision adjuvant therapy after surgery can reduce the rate of tumor recurrence and metastasis and improve the long-term survival of patients. At present, the first-line treatment drugs for advanced renal cancer are mainly tyrosine kinase inhibitors (TKIs) that target vascular endothelial growth factor receptor, such as pazopanib, sunitinib, sorafenib, axitinib, cabotinib, among others 3. Although anti-angiogenic drugs inhibit tumor proliferation to a certain extent and significantly prolong the survival of low-risk patients with ccRCC, the side effects are still obvious and the overall curative effect is not satisfactory 4, 5. In addition, even patients who are initially treated effectively will face disease progression after a certain period of time; by then, most patients will lack subsequent effective treatment.

The occurrence, growth, and metastasis of tumors are closely related with the tumor metabolism and the tumor environment 6. Tumor cells do not use mitochondrial oxidative phosphorylation, even in an aerobic environment, but instead use aerobic glycolysis. This remodeling of the energy metabolism provides tumor cells with growth and proliferation advantages, helps tumor cells escape apoptosis, and creates a tumor microenvironment (TME) that is more conducive to metastasis. When tumors occur or develop, the body may produce an adaptive immune response against tumor antigens. At the same time, immune cells infiltrating the tumor tissues could mediate an immunosuppressive TME through a variety of mechanisms to help tumor cells escape from immune recognition and attack 7. Thus, the metabolic reprogramming of tumor and immune cells in the TME is essential for understanding the biological behavior of tumor cells and the tumor immune escape and provides a new direction for regulating tumor immunity 8, 9.

In recent years, new immune checkpoint therapies (ICTs), such as PD-1/PD-L1 and CTLA4 inhibitors, have rapidly emerged in the field of renal cancer treatment, showing encouraging effects on patients with advanced refractory tumors 10. In 2020, ASCO GU announced the 5-year follow-up results of the CheckMate 025 study. The results showed that the 5-year survival rate of second-line treatment with monoclonal antibodies is as high as 26%, demonstrating the survival strengthens of ICTs 11. ICTs combined with TKIs play a role in inducing the normalization of anti-tumor immunity and in inhibiting the main signal pathways of the occurrence and development of advanced ccRCC. The success of ICTs mainly depends on deepening the understanding of the interaction between tumor cells and the TME 12. At present, ICTs combined with anti-angiogenic drugs for first-line treatment of advanced ccRCC have started a new chapter for the treatment of advanced ccRCC 13.

The Warburg effect refers to tumor cells that do not use mitochondrial oxidative phosphorylation, even under aerobic conditions. Instead, aerobic glycolysis provides up to 60% of ATP, which is identified as an important factor that leads to cancer growth promotion and metastasis 9. Increasing evidence has shown that oncogene activation, tumor suppressor gene inactivation, and TME changes affect the abnormal expression of the glucose metabolism enzymes regulating the Warburg effect 14. This remodeling of energy metabolism provides tumor cells with growth and proliferation advantages, helps tumor cells escape, and creates a microenvironment conducive to metastasis 15, 16. Tumor cells and tumor-infiltrating CD^8+^ T lymphocytes compete for glucose, and the high glucose consumption of active tumor cells changes the metabolic microenvironment of T cells and inhibits the production of IFN-γ, thus promoting tumor progression and immune escape 17. Additionally, in the TME, tumor cells and immune cells reprogram their metabolic patterns to adapt to the hypoxia, acidity, and low nutrient levels of the microenvironment 18. Therefore, exploring the mechanism of the glycolysis pathway in the immune escape and malignant progression of ccRCC has important clinical translational value for the diagnosis and prognosis of patients with ccRCC.

The hexokinase (HK) family catalyzes the conversion of glucose to glucose 6-phosphate (G6P), which is the first and rate-limiting step of anaerobic glycolysis and oxidative phosphorylation 19. The four members of the mammalian HK family (HK1-4) are similar in structure but are expressed in a tissue-specific manner. Recent studies have shown that HK1 and HK2 are significantly upregulated in many malignant tumor tissues (such as breast cancer, thyroid cancer, kidney cancer, among others), regulating the glycolysis pathways of tumor cells, and leading to a poor prognosis 20. Hexokinase-3 (HK3), as a member of the hexokinase family, is involved in the first step of glucose metabolism, and its coding gene is located on the human chromosomal 5q35.2 segment. In colorectal cancer cell lines, there is feedback regulation of the expression levels of HK1 and HK2; that is, the expression level of HK1 increases significantly after HK2 inactivation, while the expression level of HK3 is not regulated via feedback from the family members HK1 and HK2 21. The inactivation of HK3 significantly affects the activation of colorectal cancer glycolysis, and then activates the downstream signaling pathways, such as apoptosis and endoplasmic reticulum stress, in colorectal cancer cells, which plays a vital role in the progression and development of cancers 22. However, despite being a potential marker for regulating the tumor metabolic microenvironment and malignant progression, the relationship between the HK3 signal activation and progression and the ICT responses in ccRCC remains unclear.

## Materials and methods

### Ethics statement

The study design and test procedures were performed in accordance with the Helsinki Declaration II. The study protocols used in this work were approved by the ethics committee of Fudan University Shanghai Cancer Center (FUSCC, Shanghai, China).

### Raw biological microarray data

KIRC patients with available RNA sequencing data from the Cancer Genome Atlas (TCGA) database (https://tcga-data.nci.nih.gov/tcga/) were consecutively recruited for the analyses 22. The gene expression profile was measured experimentally using the Illumina HiSeq 2000 RNA Sequencing platform by the University of North Carolina TCGA genome characterization center. ESTIMATE algorithm was used to calculate the immune and stromal scores using the “Estimate” R package (http://r-forge.r-project.org; repos=rforge, dependencies=TRUE) 23.

### Patients and transcriptional expression profile from training and testing cohorts

A total of 533 ccRCC patients with available RNA sequencing data from the TCGA database were consecutively recruited for the analyses. The gene expression profiles of patients were measured experimentally using the Illumina HiSeq 2000 RNA Sequencing platform by the University of North Carolina TCGA genome characterization center. X-tile software was utilized to determine the cut-off value of the mRNA expression of *HK3*, in concordance with which all participants were divided into two groups. Student's t-test was used to compare the differential transcriptional expression levels of *HK3* between the AJCC stages or the ISUP grades, which are marked with an asterisk. The overall statistical expression difference among the AJCC stages or ISUP grades was measured using a one-way ANOVA test.

A total of 377 patients with ccRCC from the Department of Urology of FUSCC (Shanghai, China) were consecutively recruited for the analyses, from August 2009 to May 2018. Tissue samples from ccRCC and normal tissues, were collected during surgery and available from the FUSCC tissue bank.

### Gene expression omnibus (GEO) and Oncomine databases

In this study, the transcriptional *HK3* expression profiles of patients with ccRCC were obtained from the GEO database and the Oncomine online database (http://www.oncomine.com). The differences in transcriptional expression were compared using a Students' t-test. The cut-off values used for the *p*-value and fold change, among others, were as follows: *p*-value = 0.01; fold change = 1.5; gene rank = 10%; and data type: mRNA. Then, 118 ccRCC primary tumors and adjacent normal tissues from the GSE15641 (32 ccRCC samples), GSE53757 (72 ccRCC samples), and GSE66270 (14 ccRCC samples) datasets were enrolled in this study.

### Immune infiltration analysis

To investigate the association between *HK3* expression and the ccRCC immune microenvironment, the association between the abundance of infiltrating immune cells and *HK3* expression was analyzed using Tumor IMmune Estimation Resource (TIMER, https://cistrome.shinyapps.io/timer/). Additionally, TISIDB (http://cis.hku.hk/TISIDB/), an integrated repository portal of tumor immune system interactions, was also used to investigate ccRCC and the immune system interactions based on integrated multiple heterogeneous data types 24, 25.

### Survival analysis

The phenotype and expression profiles of hub genes in 533 patients with ccRCC from the TCGA were analyzed and displayed. Survival comparison between the distinct *HK3* mRNA expression groups was performed in patients with ccRCC. The primary end point for patients was progression-free survival (PFS), and the overall survival (OS) was the secondary end point, which was evaluated from the date of the first therapy to the date of death or last follow-up visit. The follow-up duration was estimated using the Kaplan-Meier method with 95% confidence intervals (95%CI) and log-rank test was used for separate curves. Univariate and multivariate analyses were performed using Cox logistic regression models to find the independent variables, including the age at diagnosis, age (reference < 60 years), gender (reference male), pT stage (reference T1-T2), pN stage (reference N0), pM stage (reference M0), AJCC stage (reference I-II), ISUP grade (reference 1-2), and *HK3* expression (reference low). X-tile software was utilized to determine the best performing thresholds.

### Processing of Gene set enrichment analysis (GSEA) data

TCGA data were analyzed using the GSEA method by using the Category version 2.10.1 package. Student's t-test statistical score was determined in consistent pathways and the mean expression of the differential genes was calculated. The operating parameters and methods were set and performed as described, respectively. The significant related genes were defined based on an adj. *p* lower than 0.01 and an FDR lower than 0.25. Statistical analysis and graphical plotting were conducted using R software (Version 3.6.1).

### Establishment of a protein-protein interaction (PPI) network and functional enrichment analysis

GeneMANIA (http://genemania.org/) online database was used for exploring and establishing a PPI network of related proteins. The interactions with a specificity score over 0.4 were considered statistically significant. Gene ontology (GO) based on the BP (biological process), and on the MF (molecular function) and KEGG pathway analyses were used to depict the functional annotations of these proteins in a bubble chart.

### Real-Time Quantitative PCR (RT-qPCR) analysis

Total RNA was extracted from 377 paired tumor and para-carcinoma normal samples using TRIzol^®^ reagent (Invitrogen Life Technologies, USA). Primers were diluted in ddH_2_O with a SYBR Green PCR Master Mix (Applied Biosystems, Japan). Transcriptional expression was determined as the fold change of *HK3* expression relative to that of *β-actin*. The PCR primer sequences used for *HK3* are as follows: forward, 5′-AGT TCT TGA CCC CAA AGA AA-3′ and reverse, 5′-TCC AAT GAC GTG TGT GCG CA-3′. The* HK3* mRNA expression was represented as ΔCt = Ct_(*HK3*)_ - ΔCt_(β-actin)_. The relative expression of *HK3* in ccRCC was represented using the ratio of *HK3* expression in tumor/normal tissues (T/N), as previously described 26. “Low *HK3* expression” and “High *HK3* expression” denote the T/N ratio of *HK3* mRNA expression with median cutoff in FUSCC cohort.

### Western blotting analysis

Total protein was extracted from cells using RIPA lysis buffer (TaKaRa) according to the manufacturer's instructions. Proteins in lysates were determined using the bicinchoninic acid (BCA) assay and 10% SDS-PAGE and then transferred onto a polyvinylidene fluoride (PVDF) membrane. The membrane was incubated with blocking buffer for 2 h at room temperature and then with the primary antibody anti-HK3 (1:1000, ab1262173, Abcam) overnight at 4 °C. Then, the protein was visualized using ECL plus western blotting detection reagents (Biosciences) and detected with an enhanced chemiluminescence kit.

### Cell culture and transfection

Human ccRCC A498 and 786O cells were cultured in DMEM. All media were supplemented with 10% FBS (v/v), 2 mM L-glutamine and 100 U/mL penicillin/streptomycin. Cells were cultured at 37 °C in a humidified atmosphere with 5% CO_2_. Both ccRCC cells A498 and 786O cells line were transfected with negative control, shRNA-1, shRNA-2 and HK3 overexpression plasmid using Lipofectamine 3000 reagent (Invitrogen) according to the manufacturer's instructions. Cells were harvested for further analysis at 24 h after transfection.

### Cell viability assay

A498 and 786O cells line were seeded into 96-well plates at a density of 3×10^3^ cells/well, and cultured in a 5% CO_2_ incubator at 37 degrees Celsius for 0 h, 24 h, 48 h, 72 h, 96 h and 120 h. Then, 10 μl CCK8 solution was added to each well, and the cells were cultured for 30 min to 4h according to the manufacturer's instructions. The OD value of the medium was detected using a spectrophotometer at 450-nm wave length.

### Cell apoptosis and cycle assays

Apoptosis detection assay was performed using Annexin V-FITC Apoptosis Detection Kits (BD, USA) using a FACS analyzer (BD, USA) in accordance with the manufacturer's experiment procedures. After A498 and 786O cells were collected and washed in PBS for three times, 500 ul cell suspension, 5 ul Annexin V-FITC, and 5 ul propidium iodide (PI) solution were resuspended in each collection tube. Flow cytometry was performed to measure the effect of different groups on cell cycle distribution of A498 and 786O cells. After A498 and 786O cells were collected and washed in PBS for three times, and disposed using cell cycle assay kit (Nanjing KeyGen Biotech), the percentage of the cells number of each cell cycle phase (G0/G1, S, G2M) was detected using a FACS analyzer (BD, USA).

### HK3 expression mapping using single-cell RNA sequencing (scRNA-seq) data

Tumor Immune Single-cell Hub (TISCH, http://tisch.comp-genomics.org/home/) is used to screen for scRNA-seq datasets with detailed cell-type annotation at the single-cell level focusing on tumor microenvironment across different cancers. GSE111360 (n=2, number of cells=23,130), GSE139555 (n=3, number of cells=49,907) and GSE145281_PDL1 (n=4, number of cells=44,220) were enrolled with correlation analyzed between HK3 expression and abundance of immune cells infiltrations.

### Statistical analysis

All statistical analyses and graphical plotting were performed with SPSS (version 23.0), GraphPad Prism 8 or R software (version 3.3.2). The Kaplan-Meier method with the 95%CI and log-rank test were used in separate survival curves. A two-tailed Student's t-test or one-way ANOVA was used to measure differences between groups. All hypothetical tests were two-sided and *p*-values less than 0.05 were considered statistically significant in all tests.

## Results

### Identification of differential expressed HK3 in ccRCC and normal samples from multiply cohorts

**Figure 1:**
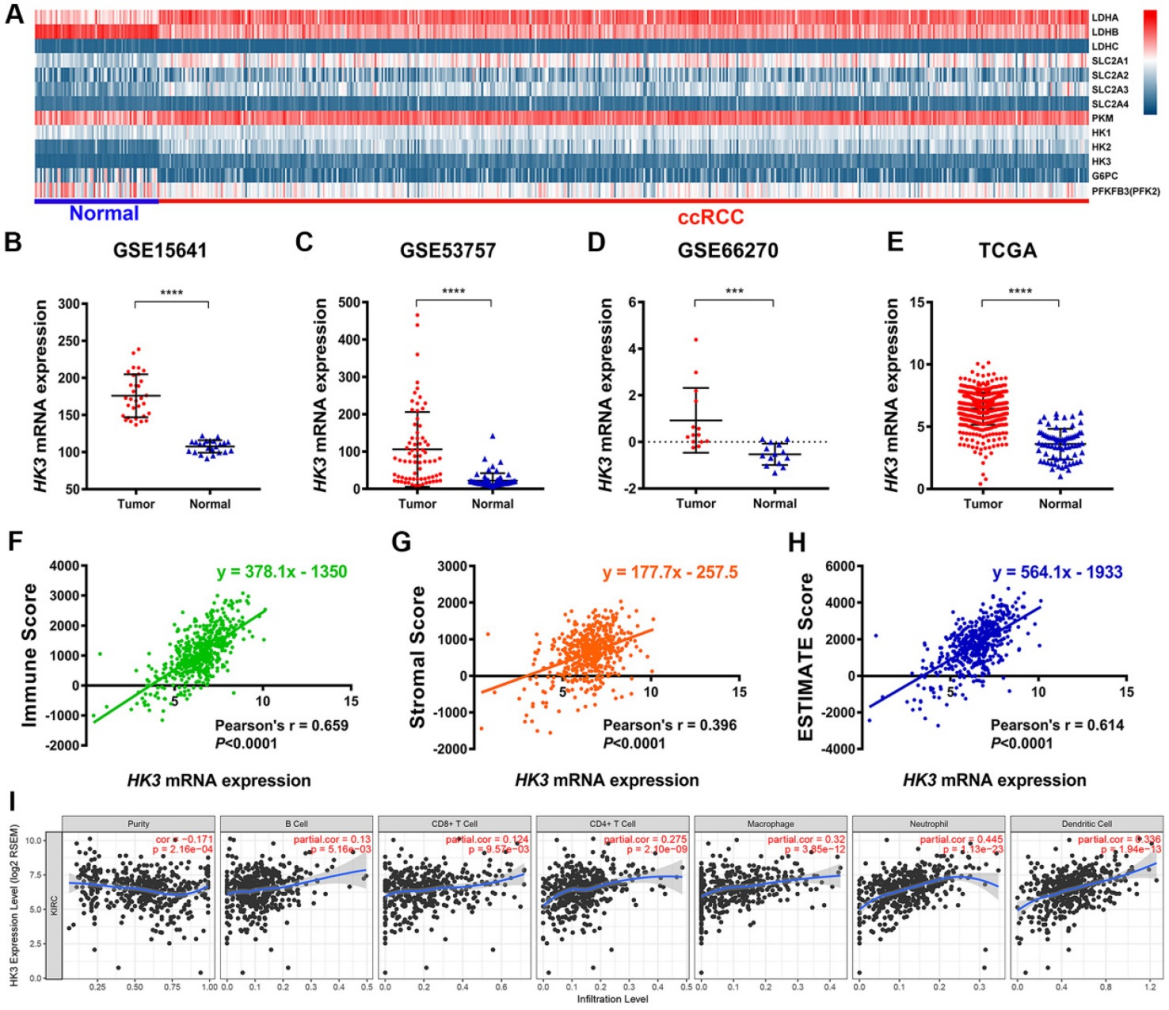
** Identification of the differential HK3 expression in ccRCC and normal samples and its correlation with immune cell infiltration based on multiple cohorts.** (**A**) Differential expression analysis of hub genes related to the glycolysis signaling pathway, including *HK1-3*, *LDHA-C*, *SLC2A1-4*, *PKM*, *G6PC*, and *PFKFB3*, between ccRCC tissues and adjacent normal tissues in 533 ccRCC patients from the TCGA cohort. (**B-D**) Differential *HK3* expression was observed in 118 ccRCC primary tumors, in comparison with the adjacent normal tissues from GSE15641 (32 ccRCC samples), GSE53757 (72 ccRCC samples), and GSE66270 (14 ccRCC samples), as per Student's t-test. (**E**) The transcriptional expression levels of *HK3* were significantly high in 533 ccRCC tissues, compared with 72 normal tissues in the TCGA-KIRC cohort. (**F-H**) Spearman's correlation indicated a relationship between *HK3* and the immune contexture, stromal contexture, and the tumor purity of the ccRCC microenvironment, using ESTIMATE algorithm. (**I**) The association between the abundance of infiltrating immune cells and *HK3* expression was analyzed.

Explore the mechanism of glycolysis pathway in immune escape and malignant progression carcinoma has important reference value for the diagnosis and prognosis of ccRCC patients. We first identified differential expression of hub genes related to glycolysis signaling pathway, including HK1-3, LDHA-C, SLC2A1-4, PKM, G6PC and PFKFB3, between ccRCC tissue and adjacent normal tissues were compared in 533 ccRCC patients from TCGA cohort **(Figure 1A).** It suggested that hexokinases (HKs) family were highly expressed in tumor samples compared with normal samples. Then, we focused on role of *HK3*, and found *HK3* expression was significantly higher in 118 ccRCC primary tumors in comparison with adjacent normal tissues in GSE15641 (32 ccRCC samples, ****, *p*<0.001) 28, GSE53757 (72 ccRCC samples, ****, *p*<0.001) 29 and GSE66270 (14 ccRCC samples, ***, *p*<0.01) 30** (Figure 1B-D)**. Transcriptional level of *HK3* expressions were significantly highly expressed in 533 ccRCC tissues compared with 72 normal tissues (****, *p*<0.0001) in TCGA-KIRC cohort **(Figure 1E).**

### HK3 expression correlated with abundance of immune cell infiltrations

Interestingly, after ESTIMATE algorithm was processed, immune scores, stromal scores and tumor purity of ccRCC were obtained. Spearman's correlation indicated strong relationship between *HK3* and immune contexture (r=0.659, *p*<0.001), stromal contexture (r=0.396, *p*<0.001) and tumor purity (r=0.614, *p*<0.001) of ccRCC microenvironment **(Figure 1F-H).** To investigate association between *HK3* and ccRCC immune microenvironment, association between abundance of immune cell infiltrations and *HK3* expression was analyzed. *HK3* expression were significantly correlated with B cell, CD8^+^ T cell, CD4^+^ T cell, and especially macrophage (cor.=0.275), neutrophil (cor.=0.445), and dendritic cell infiltration (cor.=0.336), prompting a general activation in immune microenvironment of ccRCC **(Figure 1I).**

### Prognostic implications of HK3 in cancers

**Figure 2:**
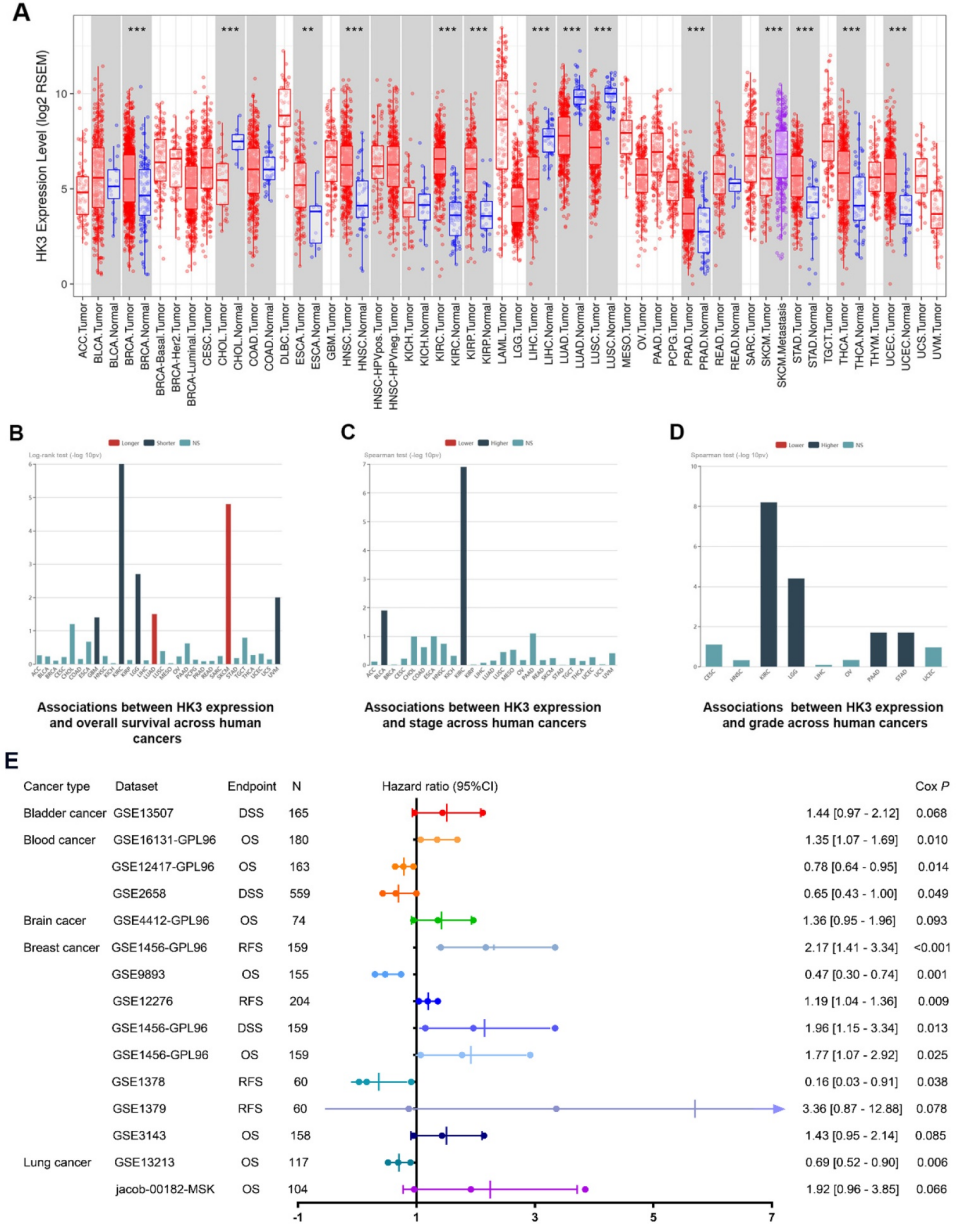
** Prognostic implications of HK3 in large-scale pan-cancer samples.** (**A**) Differential expression of HK3 in pan-cancers from the TCGA database. (**B-D**) Prognostic value of HK3 expression and its association with the stage and grade in 12,452 pan-cancer samples. (**E**) Furthermore, we enrolled 2,476 patients with bladder, blood, brain, breast, and lung cancer with available clinical data from the GEO datasets and performed Cox regression analysis of the overall survival.

In order to further demonstrate our hypothesis, we analyzed the expression of HK3 of all cancers **(Figure 2A).** Although the expression level of HK3 were significantly higher in normal tissues than in tumor tissues in CHOL, LIHC, LUAD, LUSC cohorts, *HK3* expression is mostly highly expressed in tumors compared with normal sample, and the difference is more prominent in ccRCC. At the same time, we investigate prognostic role of HK3 in various cancers. It suggested that high expression of HK3 is significantly associated with the deteriorated outcomes, increased stage and increased of grade in 12,452 pan-cancer samples **(Figure 2B-D).** Furthermore, we enrolled 2,476 patients of bladder, blood, brain, breast and lung cancer with available clinical data from GEO datasets **(Figure 2E).** Cox regression analysis suggested that *HK3* expression has closely positive association with poor prognosis. Overall, these large-scale findings highly suggested the predictive efficacy of *HK3* for tumor progression and poor prognosis.

### HK3 expression correlated with advanced clinicopathological parameters for ccRCC patients in TCGA cohort

**Figure 3:**
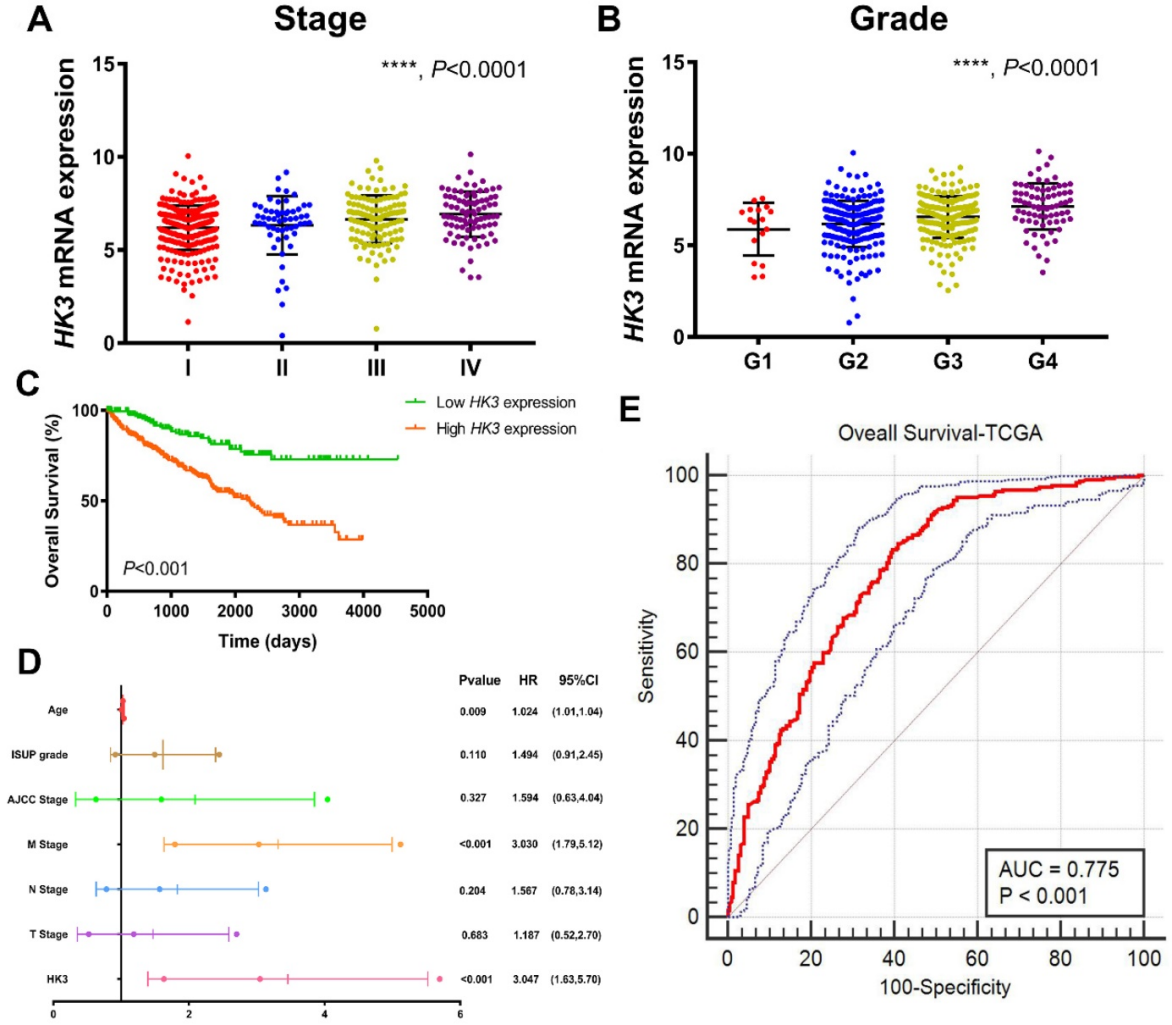
** HK3 expression was correlated with advanced clinicopathological parameters and the outcomes of ccRCC patients in the TCGA cohort.** (**A-B**) *HK3* mRNA expression in ccRCC samples was significantly associated with advanced clinical stages and the pathological grade, as shown using ANOVA test. (**C**) Kaplan-Meier method showed that an elevated *HK3* expression was significantly correlated with a shorter OS in 533 ccRCC patients from the TCGA cohort. (**D**) In multivariate Cox regression analysis, traditional prognostic factors, specifically the pM stage, were shown to still be relevant to OS in ccRCC patients from the TCGA cohort. (**E**) After integrating all the significant clinicopathological parameters and gene expression profiles in the Cox regression models, we generated the following formula: 1.024 × Age + 3.030 × pM stage (reference M0) + 3.047 × HK3 expression (reference low) for OS. The AUC index of the TCGA-OS was 0.775, highlighting the accuracy and consistency of the predictive power of *HK3* in ccRCC.

After integrating clinicopathological and survival data from TCGA, significantly elevated* HK3* mRNA expression was found in ccRCC samples compared with normal samples. As was shown in **Figure 3A,** *HK3* mRNA expressions of ccRCC samples were significantly associated with advanced clinical stages (*p*<0.001), and the highest *HK3* mRNA expressions were found in stage 4. In **Figure 3B**, relationship between *HK3* mRNA expression and different pathological grade was measured, which suggested that mRNA expressions of *HK3* were significantly correlated with pathological grades (*p*<0.001). Similarly, the highest *HK3* mRNA expressions were found in grade 4. Overall, elevated *HK3* mRNA expression was significantly associated with advanced clinicopathological parameters of ccRCC patients.

After identifying differentially expressed *HK3* expression, Kaplan-Meier method showed that elevated *HK3* expression was significantly correlated with shorter OS (*p*<0.001) in 533 ccRCC patients from TCGA cohort **(Figure 3C).** Univariate Cox analysis suggested pathological TNM stage, AJCC stages, ISUP grade and HK3 expression as prognostic indicators in 533 ccRCC patients **(Supplementary Figure 1).** In multivariate Cox regression analysis, traditional prognostic factors, specifically pM stage, were still relevant to OS (HR=3.030, *p*<0.001) in ccRCC patients from TCGA cohort **(Figure 3D).** In addition, age was significant in OS (age:* p*=0.009). After integrating all the significant clinicopathological parameters and gene expression profiles in the Cox regression models, we generated the formula= 1.024×Age + 3.030×pM stage (ref. M0) + 3.047×HK3 expression (ref. Low) for OS. The AUC index for the TCGA-OS were 0.775 (*p*<0.001; **Figure 3E**), significantly higher than the predictive power of traditional clinicopathological factors and other models, highlighting the accuracy and consistency of *HK3* in the predictive power of ccRCC.

### Subgroup survival analysis indicated HK3 expression correlated with poor prognosis for ccRCC patients

**Figure 4:**
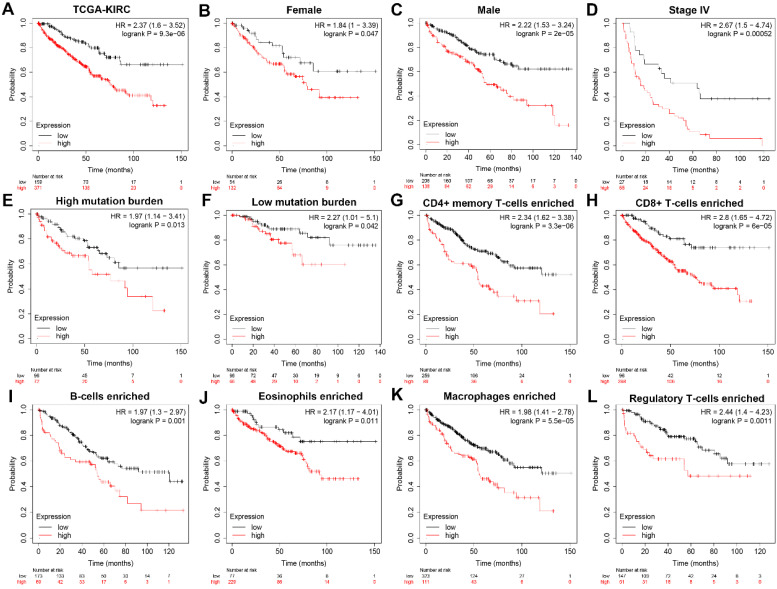
** Subgroup survival analysis indicated that HK3 expression is an independent prognostic biomarker for ccRCC patients.** (**A**) To further explore whether *HK3* can be used as an independent predictor, subgroup survival analysis was performed in all ccRCC patients from the TCGA-KIRC cohort. (**B-L**) We grouped all patients according to their gender, stage, mutation burden, and immune cell enrichment, and found that patients with a high *HK3* expression had a significant decrease in the survival rate, an increased tumor stage, and increased immune cell infiltration.

To further explore whether *HK3* can be used as an independent predictor, subgroup survival analysis was performed. We grouped all patients according to their gender, stage, mutation burden, immune-cells enriched, and found that patients with high *HK3* had a significant decrease in survival rate, increased tumor stage, and increased immune cell infiltration (*p*<0.01). It shows that *HK3* serves as a promising and potential tumor marker predicting prognosis and survival of ccRCC patients **(Figure 4).**

### HK3 mRNA expression correlated with advanced clinicopathological parameters for ccRCC patients in FUSCC cohort

**Figure 5:**
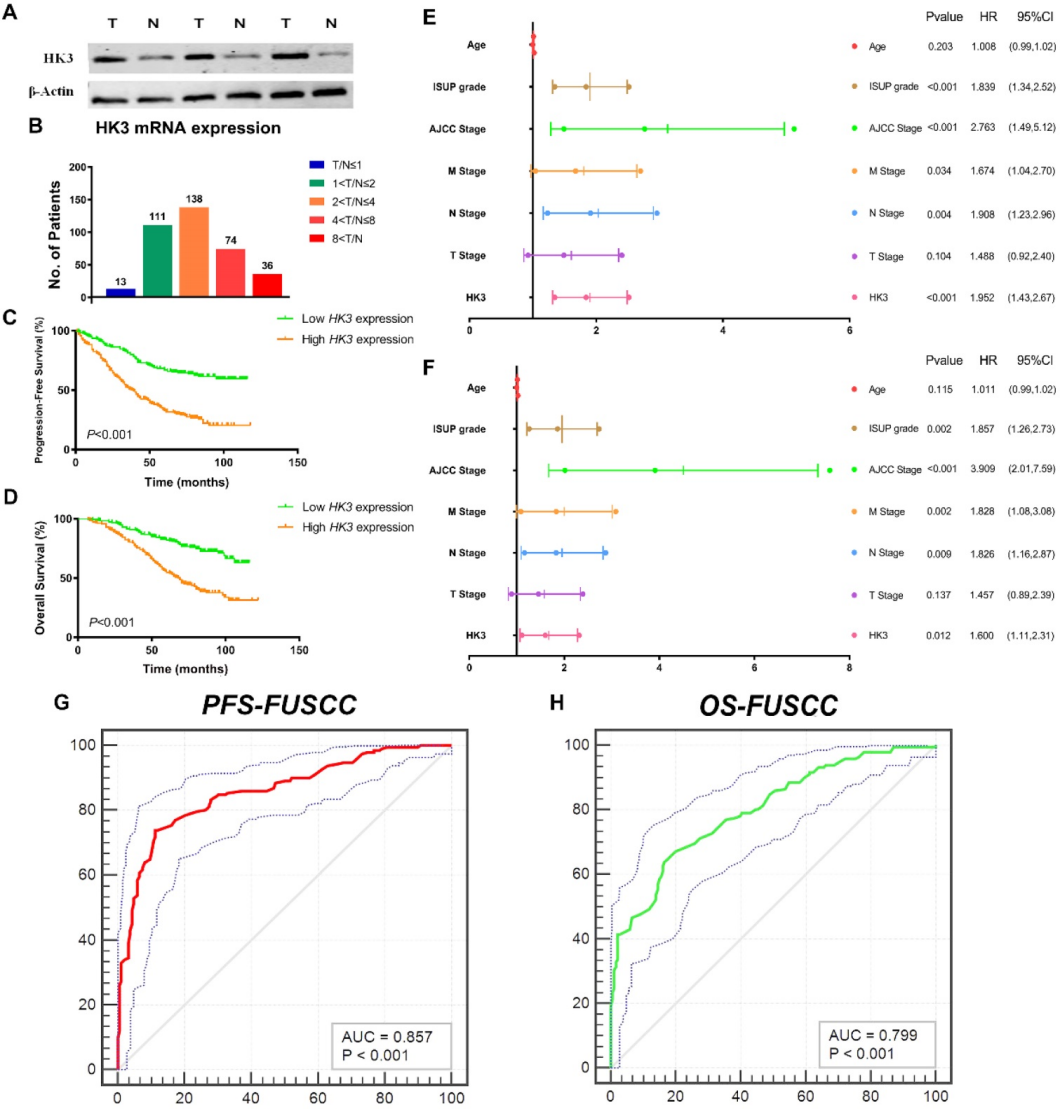
** HK3 mRNA expression was correlated with advanced clinicopathological parameters and a poor prognosis in ccRCC patients from the FUSCC cohort.** (**A**) To validate the differential *HK3* expression profile prognostic implications *in vitro*. We measured the HK3 expression level in three paired ccRCC tumor and normal samples from the FUSCC cohort. (**B**) We performed RT-qPCR using 377 paired tumor and normal samples with available clinical follow-up data from the FUSCC cohort. The HK3 mRNA expression was represented as ΔCt = Ct(*HK3*) -ΔCt(*β-actin*). The relative expression in ccRCC was represented using the ratio of *HK3* expression in tumor/normal tissues (T/N). A low HK3 expression and a high HK3 expression denote the T/N ratio of *HK3* mRNA expression based on the median cut-off in the FUSCC cohort. (**C-D**) Survival curves suggested the prognostic value of *HK3* mRNA expression in 377 ccRCC patients from the FUSCC cohort using Kaplan-Meier method. (**E-F**) In multivariate Cox regression analysis, *HK3* amplification was markedly correlated with a poor PFS and a poor OS using forest plots. (**G-H**) After integrating all the significant clinicopathological parameters and gene expression profiles in the Cox regression models, we generated the formulas for PFS and OS.

To validate differential *HK3* expression profile prognostic implications *in vitro*. We measured HK3 expression level in three paired ccRCC tumor and normal samples from FUSCC cohort. Significantly elevated *HK3* expression in human ccRCC tissues was found compared with normal tissues in protein levels **(Figure 5A).** In addition, we performed RT-qPCR using 377 paired tumor and normal samples with available clinical follow-up data from FUSCC cohort. It revealed dramatically increased *HK3* mRNA expression in ccRCC samples that 96.6% of patients had higher levels of *HK3* expression in tumor tissues than normal tissues **(Figure 5B).** Survival curves suggested that with elevated *HK3* mRNA levels significantly correlated with poorer PFS and OS in 377 ccRCC patients from FUSCC cohort (*p*<0.001; **Figure 5C-5D**).

In univariate Cox regression analysis models, traditional prognostic factors such as pTNM stage, AJCC stage, and ISUP grade were significantly relevant to PFS (*p*<0.001) and OS (*p*<0.001) for ccRCC patients in FUSCC cohort, demonstrating a fine representativeness of the population **(Supplementary Table 1).** *HK3* amplification markedly correlated with poor PFS (HR=2.852, *p*<0.001) and poor OS (HR=2.999, *p*<0.001) In addition, pM stage, pN stage, AJCC stage and ISUP grade were significant both in PFS (pM stage:* p*=0.034, pN stage:* p*=0.004, AJCC stage: *p*<0.001, ISUP grade: *p*<0.001) and OS (pM stage:* p*=0.002, pN stage:* p*=0.009, AJCC stage:* p*<0.001, ISUP grade: *p*=0.002) based on FUSCC cohort **(Figure 5E-5F).** Importantly, elevated *HK3* mRNA expression was significantly associated with poor PFS (HR=1.952, *p*<0.001) and poor OS (HR=1.600, *p*=0.012) in FUSCC cohorts for ccRCC patients.

After integrating all the significant clinicopathological parameters and gene expression profiles in the Cox regression models, we generated the formula= 1.782×pT stage (ref. T1-T2) + 1.937×pN stage (ref. N0) + 1.763×pM stage (ref. M0) + 2.425×AJCC stage (ref. I-II) + 1.812×ISUP grade (ref. 1-2) + 1.714×HK3 expression (ref. Low) for PFS, and another formula= = 1.692×pT stage (ref. T1-T2) + 1.837×pN stage (ref. N0) + 1.895×pM stage (ref. M0) + 3.553×AJCC stage (ref. I-II) + 1.751×ISUP grade (ref. 1-2) + 1.514×HK3 expression (ref. Low) for OS. The AUC index for the FUSCC-PFS and FUSCC-OS were 0.857 and 0.799, respectively (*p*<0.001; **Figure 5G-5H**).

### Transcriptional expressions of HK3 and clinicopathological characteristics were balanced on the distribution of categorical data in two cohorts

In order to further verify the ability of HK3 as a tumor marker of ccRCC, we collected clinicopathological characteristics baseline in relation to HK3 mRNA expression status in 910 ccRCC patients from TCGA and FUSCC cohorts. As shown in **Table 1,** ccRCC patients with increased HK3 mRNA expression significantly correlated with advanced pT (*p*<0.001), pN (*p*<0.001), pM stage (*p*<0.001), AJCC stage (*p*<0.001) and ISUP grade (*p*=0.026) in FUSCC cohort. In addition, In TCGA cohort, increased HK3 mRNA expression significantly correlated with advanced pT (*p*=0.007), pM stage (*p*=0.04), AJCC stage (*p*=0.005) and ISUP grade (*p*<0.001).

### HK3 regulates cell proliferation and affects glycolysis

**Figure 6:**
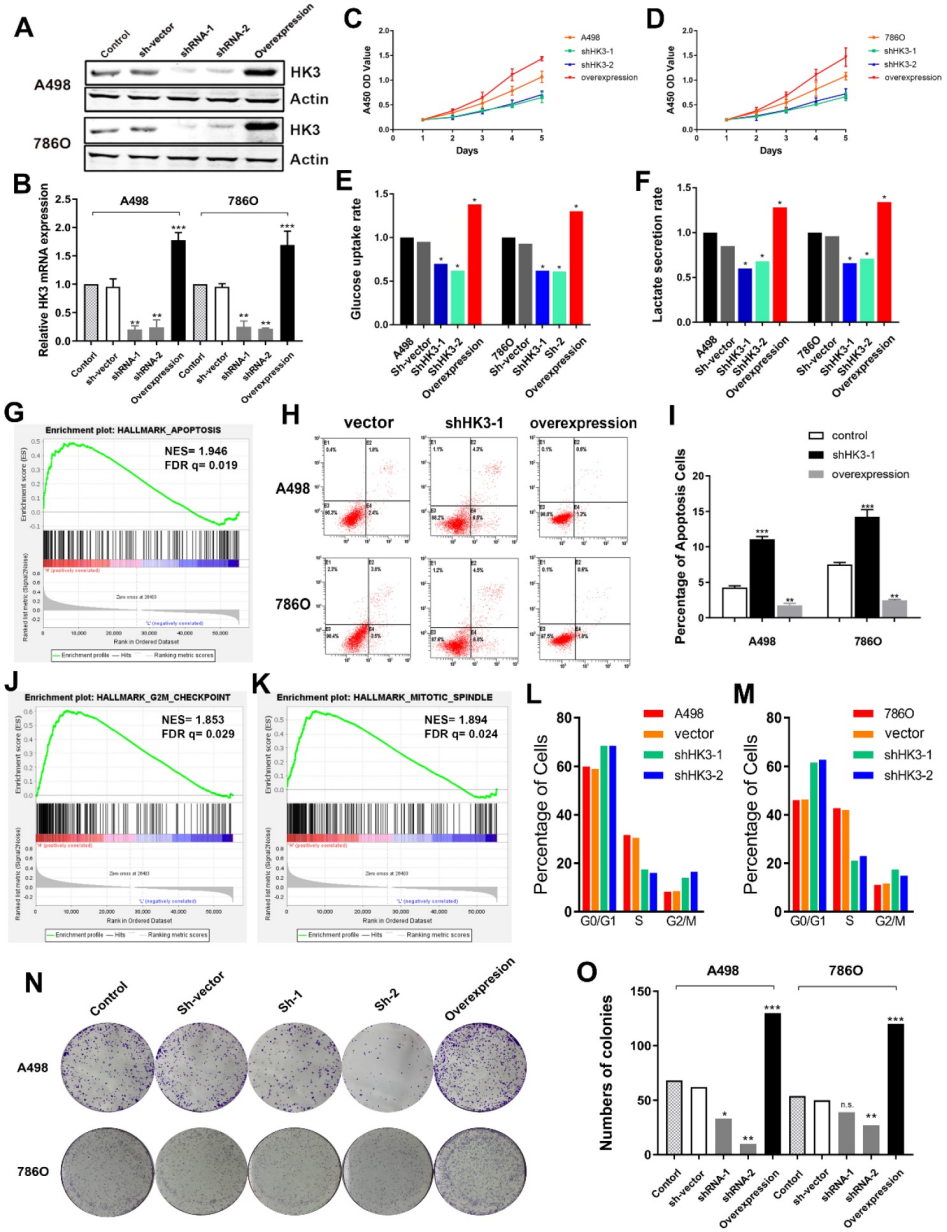
** HK3 regulates malignant biologic behaviors in A498 and 786O cells.** (**A**) The expression level of *HK3* was significantly decreased in the shRNA-1-transfecting group, the shRNA-2-transfecting group cells, and significantly increased in the *HK3* overexpression-transfecting group in A498 and 786O cells. (**B**) The transcriptional *HK3* expression was also significantly decreased in the shRNA-1 and shRNA-2 groups, compared with the normal control, and elevated in the overexpression group, as shown using ANOVA test. (**C-D**) CCK8 analysis suggested that the cell growth value of the HK3 shRNA groups was significantly decreased compared to the normal control group. (**E-F**) HK3 affected the glucose uptake and lactate secretion in other treated groups, compared with the vector group, as shown using an unpaired t-test. (**G**) GSEA was used to explore the involved hallmarks that had changed by *HK3* expression changes. (**H-I**) *HK3* overexpression significantly decreased the number of apoptotic cells, compared with the shRNA-1 cell group, as measured using propidium iodide (PI) and FITC‐Annexin V fluorescence. (**J-K**) GSEA suggested that the most significant pathways involved included the G2/Mcheckpoint and mitotic spindles. (**L-M**) Cell cycle assay indicated that there were more cells in the G0/G1 and G2/M phases and less cells in the S phase, compared with the negative control. (**N-O**) Colony formation assay indicated that the inhibition of *HK3* significantly decreased the proliferation, and the overexpression of *HK3* significantly increased the proliferation ability of human ccRCC cells.

Subsequently, we explored whether *HK3* could modulate the malignancy of ccRCC cell. We investigated the expression level of* HK3* in A498 and 786O cells after transfection with sh-vector, shRNA-1, shRNA-2 or overexpression. The results demonstrated that the expression level of *HK3* was significantly decreased in shRNA-1-transfecting group, shRNA-2-transfecting group cells, and significantly increased in the *HK3* overexpression-transfecting group in A498 and 786O cells **(Figure 6A).** Similarly, transcriptional *HK3* expression was also significantly decreased in shRNA-1, shRNA-2 groups compared with normal control, and elevated in overexpression group **(Figure 6B).** Next, to detect the potential function of *HK3*, we assessed cell proliferation using a CCK-8 assay after transfecting shRNA-1, shRNA-2 or overexpression into A498 and 786O cells. After being up-regulated or down- regulated of *HK3* by shRNA-1, shRNA-2 or overexpression, the cell growth value of HK3 shRNA-1 group was significantly decreased compared to the normal control group **(Figure 6C-D).** Next, we found that *HK3* affects glucose uptake and lactate secretion. *HK3* overexpression significantly increases glucose uptake and lactate secretion. Conversely, *HK3* knockdown can inhibit glucose uptake and lactate secretion **(Figure 6E-F).**

### HK3 regulates cell apoptosis, cell cycle and clone formation

Then, GSEA was used to explore involved hallmarks changed by interference of *HK3* expression. First, the results suggested that HK3 significantly participated in apoptosis pathways of ccRCC (NES=1.946, FDR q=0.019; **Figure 6G**). As shown in **Figure 6H-I,** after transfection with *HK3* shRNA-1 or overexpression in A498 and 786O cells, we found that *HK3* overexpression significantly decreased apoptosis cells compared to shRNA-1 cell groups measured by propidium iodide (PI) and FITC ‐ Annexin V fluorescence. In addition, GSEA also suggested that the most involved significant pathways included G2/Mcheckpoint (NES=1.853, FDR q=0.029) and mitotic spindle (NES=1.894, FDR q=0.024) **(Figure 6J-K).** To further measure effect of *HK3* in cell cycle, we transfected shRNA of *HK3* into A498 and 786O cells, and found increased in G0/G1 and G2/M phases and decreased in S compared with the negative control **(Figure 6L-M).** Meanwhile, Colony formation assay indicated that inhibition of *HK3* significantly decreased the proliferation, and overexpression of *HK3* significantly increased the proliferation ability of human ccRCC cells (**Figure 6N-O**).

### HK3 correlated with monocyte/macrophage infiltration and lipid metabolism in ccRCC microenvironment

**Figure 7:**
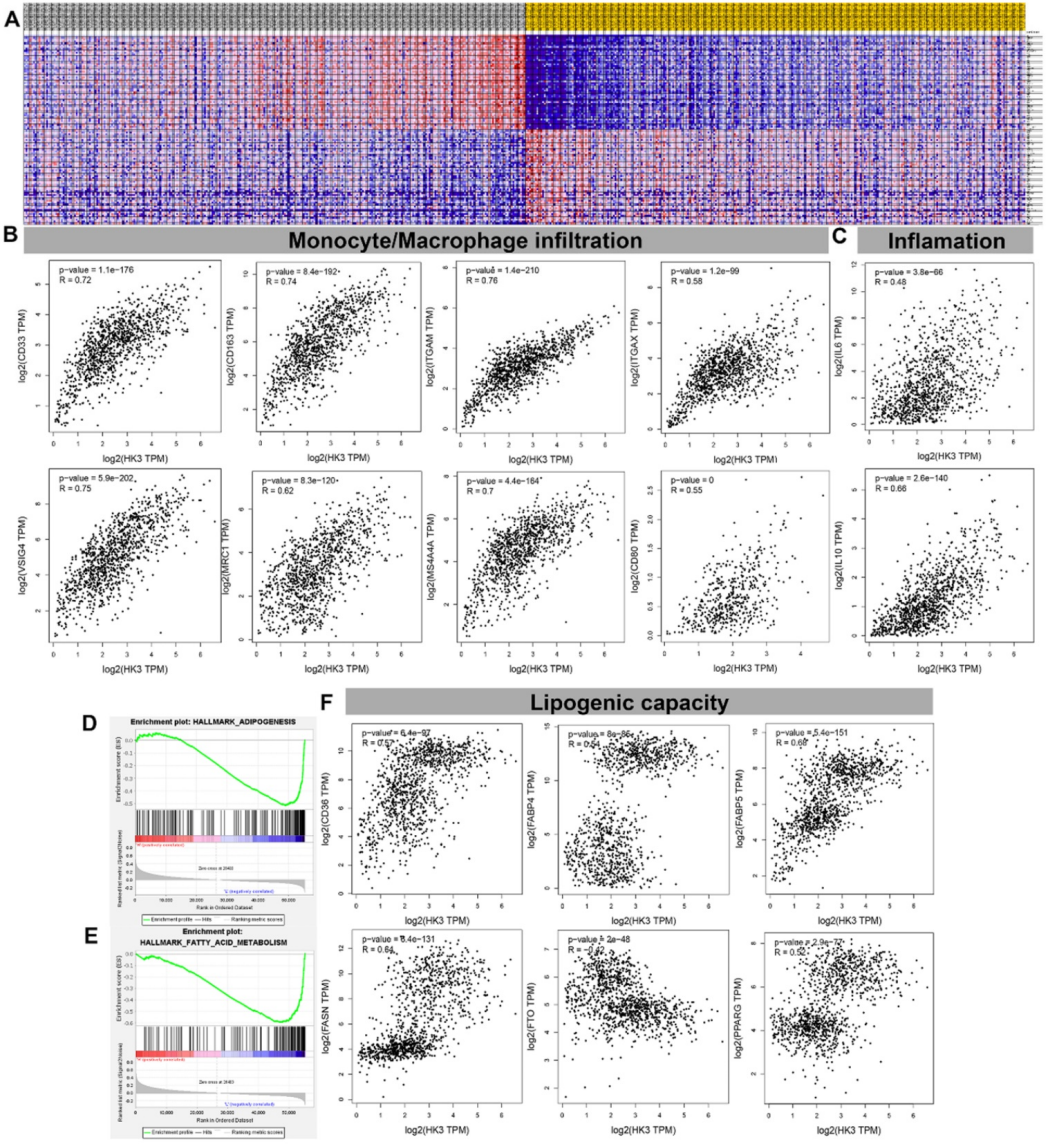
** HK3 was correlated with monocyte/macrophage infiltration and lipid metabolism in the ccRCC microenvironment.** (**A**) GSEA analysis showed the transcriptional expression profiles of the 100 significant genes that were positively and negatively correlated in a heat map. (**B-C**) After combining the data of the ccRCC tissues, adjacent normal tissues from the TCGA database, and visceral, subcutaneous adipose tissue from the GTEx database, we explored the relationship between *HK3* activation and horizontal monocyte/macrophage infiltration, inflammatory infiltration, and lipid formation processes using Pearson's correlation test. (**D-E**) GSEA was used to explore the involved fatty acid metabolism-related hallmarks that changed by the *HK3* expression alterations. (**F**) The degree of *HK3* activation was related with the intracellular downstream signals involved in lipid synthesis and with CD36, FABP5, FASN, FTO, and other markers that mediate the lipid formation process and lipid droplet transport in kidney tissues, as shown using Pearson's correlation test.

GSEA analysis showed transcriptional expression profiles of the 100 significant genes positive and negative correlation in a heat map** (Figure 7A).** In order to further study and analyze the differences in the immune molecular infiltration of the ccRCC microenvironment mediated by *HK3*, we combined the data of ccRCC tissues, adjacent normal tissues from TCGA database, as well as visceral and subcutaneous adipose tissue from GTEx database, to explore the relationship between *HK3* activation and horizontal monocyte/macrophage infiltration, inflammatory infiltration and lipid formation processes. It indicated that the expression level of *HK3* is closely related to the infiltration level of a large number of immune cell surface markers in the microenvironment of ccRCC patients *in silico*. In tumor environment of activated ccRCC, the proportion of CD86^+^CSF1R^+^ monocytes were significantly increased. Also, the surface molecules of M2 macrophages involved in secretion of lipids, such as CD163, VSIG4, MS4A4A, ITGAM and ITGAX, proportion of inflammation cells infiltration significantly increased with elevated HK3 expression **(Figure 7B-C).** In addition, Spearson's correlation test revealed a series of immune cells and surface markers infiltration levels related to the high activation of *HK3* in the ccRCC microenvironment (the correlation coefficient is greater than 0.4, P value less than 0.0001), such as monocyte surface markers CD86, CD115, tumor-associated macrophage markers CD68, IL10, M2 macrophage markers CD163, VSIG4, MS4A4A, neutrophil markers CD11b (ITGAM) and etc. **(Table 2).** In **Figure 7D- F,** the degree of *HK3* activation is not only closely related to the infiltration level of CD33^+^ CD163^+^ tumor-associated monocytes, VSIG4^+^ M2 type macrophages, but also correlated with the intracellular downstream signals involved in lipid synthesis and CD36, FABP5, FASN, FTO and other markers that mediate lipid formation process and lipid droplet transport in kidney tissues.

### Functional enrichment analysis of HK3 and related genes

**Figure 8:**
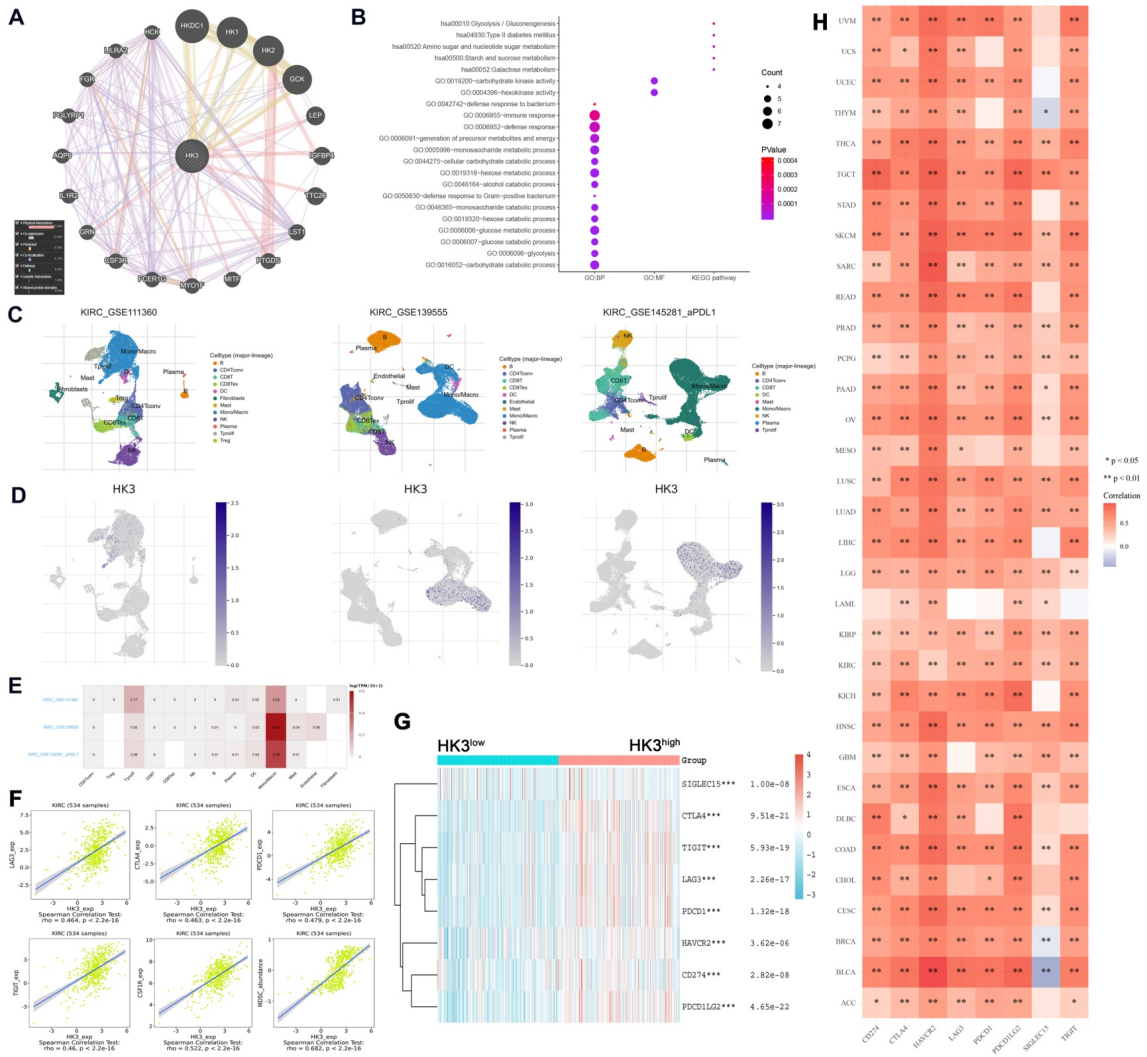
** HK3 was correlated with immune checkpoint molecules and active anti-tumor immune escape via regulating monocyte/macrophage infiltration in cancers.** (**A**) Gene-gene and protein-protein interactions of *HK3* and 20 related genes was performed. Different line colors represent different types of gene-gene interaction networks. (**B**) GO and KEGG functional analysis were used to predict the pathways in which HK3 participates. (**C-D**) Three single-cell RNA-seq datasets (GSE11136, GSE13955, and GSE145281) were enrolled to determine the location of HK3 in different cell types. (**E**) We quantitatively calculated the positioning and binding of HK3 on various immune cells across the dataset using a heatmap. (**F**) Correlation analysis showed the association between the expression of HK3 and that of immune checkpoint molecules, including LAG3, CTLA4, PDCD1, TIGIT, and CSF1R (rho > 0.4), and the abundance of MDSCs, using Spearson's correlation test. (**G**) Immune checkpoint-related gene expression heatmap, where different colors represent the expression trends in different samples. The asterisk represents the degree of importance (*p). The significance of the two groups of samples passed the Wilcox test. (**H**) Heatmaps of the expression of HK3 in tumor tissues, where the horizontal axis represents the different immune checkpoint genes, and the vertical axis represents the various tumor tissues (*p < 0.05, **p < 0.01, and ***p < 0.001).

As illustrated in **Figure 8A**, gene-gene protein interaction of *HK3* and related 20 genes was performed. Different line colors represent different types of gene-gene interaction networks. 67.64% terms were in physical interactions (pink line), 13.50% terms were in co-expression (purple line), 6.35% terms were predicted (khaki line), 6.17% terms were co-localization (blue line), 4.35% were in pathway (sky blue line), 1.40% terms were in genetic interactions (green line) and 0.59% shared protein domains (yellow line). In the GO and KEGG pathways, we found that *HK3* is closely related to glycolysis/gluconeogenesis, defense response to bacterium, carbohydrate kinase activity and immune responses (**Figure 8B**).

### HK3 correlated with immune checkpoint molecular via regulating monocyte/macrophage infiltration

In order to reduce the heterogeneity of tumor tissues, and to further confirm localization of *HK3* in immune cells, we included and analyzed three single-cell RNA-seq (scRNA-seq) datasets, GSE11136, GSE13955 and GSE145281. As shown in **Figure 8C-D**, HK3 is mainly located or bind to monocyte/macrophage. A heatmap showed the relatively high expression of a *HK3* in different cell types across three scRNA-seq datasets (**Figure 8E**). As shown in **Figure 8F**, it can be seen that HK3 has a significant correlation with many immune checkpoint molecules, including LAG3, CTLA4, PDCD1, TIGIT and CSF1R (rho>0.4), and abundance of MDSC (rho=0.682) using Spearson's correlation test. Next, siglec15, IDO1, CD274, HAVCR2, PDCD1, CTLA-4, LAG-3, and PDCD1LG2 were selected to be immune-checkpoint-relevant transcripts and *HK3* expression values of these eight genes were extracted in **Figure 8G**, it suggested that the immune checkpoint molecules expression was significantly elevated win high *HK3* expression group compared with HK3^low^ group of ccRCC.

### HK3 may participate in tumor immune microenvironment remodeling and predict ccRCC patients receiving ICTs

Next, to explore whether HK3 involved in immune-rejection microenvironment of all cancers, we extracted the expression values of siglec15, IDO1, CD274, HAVCR2, PDCD1, CTLA-4, LAG-3, and PDCD1LG2 in totally 10,201 multiple tumor tissues (**Figure 8H**). Spearman correlation analysis heat map showed that the eight genes significantly correlated with *HK3* mRNA expression, prompting the microenvironmental characteristics of active anti-tumor immune responses. After proving that HK3 is related to the immune environment of ccRCC, we further studied the role of *HK3* in pan-cancer. On the one hand, we calculated the ESTIMATEScore, ImmuneScore and StromalScore of HK3 in various cancers. In **Figure S2A**, *HK3* plays an important role in the immune microenvironment of various cancers. In order to further study the role of *HK3* in immunotherapy, we found high relationship between it and tumor neoantigens, especially prostate cancer and colon cancer (**Figure S2B**). At the same time, we also studied the relationship between *HK3* and various immune checkpoint factors and abundance of immune cells. In **Figure S2C-D**, *HK3* significantly correlated with various surface molecules of immune cells and immune checkpoint molecules (*p*<0.05).

## Discussion

As the main type of malignant kidney cancer, ccRCC has a significant feature at the chromosome level, that is, 90% of patients have changes in the short arm of chromosome 3 27. A series of tumor suppressor genes, such as VHL, PBRM1, BAP1 and STED2, were located on the short arm of chromosome 3 and are responsible for regulating the occurrence and development of ccRCC 27. As the most frequent mutations, Von Hippel-Lindau (VHL) encoded protein pVHL and ubiquitinate hypoxia inducible factor (HIF), thereby promoting the degradation of HIF 28, 29. Under hypoxic environment, HIF could initiate the expression of a large number of downstream regulatory factors, such as glucose transporter (GLUT), vascular endothelial growth factor (VEGF), transforming growth factor β (TGF-β) and epidermal growth factor (EGF), etc., thereby leading to unique highly active energy metabolism mode of ccRCC 30. The fixed mode of mitochondrial respiration is changed to aerobic glycolysis, which promotes angiogenesis, changes the immune microenvironment, and is conducive to tumor proliferation, invasion, metastasis, epithelial-mesenchymal transition and immune escape.

A large number of studies have confirmed that the increased glucose metabolism caused by glycolysis promotes the growth, survival, proliferation and long-term maintenance of tumor cells, and is an important sign of cancer progression and deterioration 31. As the first rate-limiting enzyme for sugar decomposition and energy, hexokinases (HKs) could convert glucose into glucose-6-phosphate. Glucose-6-phosphate, as an intermediate product of multiple substance synthesis pathways, promotes the progression and malignant behaviors of cancers 19. Therefore, it is of great significance to inhibit HKs in the process of energy supply of malignant tumor tissues. Previous studies found that HK3 plays a major role in acute promyelocytic leukemia and colorectal cancer, while the underlying mechanism and its role in TME remains to be elucidated 32-34. In this study, significantly prognostic implications of HK3 has been identified in ccRCC based on multiply cohorts (n>1,500) and in many cancers. Besides involved in glycolysis metabolism of ccRCC microenvironment, *HK3* promotes malignant cell behaviors. Normally, tumor glycolysis activation promotes anti-tumor immune responses, elevated immune check-point molecules (PD-L1) expression levels, and thus imposed a better ICT response in cancers 35. Thus, the association between metabolic reprogramming, specifically glycolysis, and TME of ccRCC worth further investigation.

ICTs combined with TKI play a role in inducing the normalization of anti-tumor immunity, inhibiting the main signal pathways of the occurrence and development of advanced ccRCC 36, 37. With the deepening of research, more evidence has shown that not only the efficacy of ICTs depends on the activation of the tumor immune microenvironment, but the efficacy of traditional treatment methods, such as targeted therapy, also depends on the strength of the anti-tumor immune response 38, 39. The cells and molecules in the TME are in a process of dynamic change, reflecting the evolutionary nature of cancer, and jointly promoting immune escape, and the growth and metastasis of tumors. Therefore, exploring the underlying mechanism of TME-driven tumorigenesis and its development is of great significance for developing potential methods of cancer treatment, improving the effectiveness of various existing treatment methods, and discovering new precise targets for ccRCC treatment. This study found that HK3 stimulates the infiltration of monocytes/macrophages presenting surface markers, regulates the key molecules PD-1 and CTLA-4 of exhaustive T cells, and affects the immune escape process of cancers. These findings provided novel insights into HK3 regulation of the activation of immune cells in the tumor microenvironment to mediate immune escape and provides a new theoretical basis for understanding the metabolic network of the ccRCC microenvironment and discovering new therapeutic targets.

The advantage of this study is that we first assessed the prognostic implications of the differential expression levels of HK3 in ccRCC and pan-cancers based on large-scale population cohorts. Second, this study focuses on the diversity and complexity of the immune cells infiltrated in the tumor microenvironment of ccRCC and aimed to assess three aspects: the identification of specific immune cell subgroups, functional phenotype analysis, and investigating the underlying mechanism. Cancer-promoting HK3 signal mediates the infiltration of monocytes/macrophages and promotes tumor immune escape. Third, this study performed complex bioinformatics methods, including immune infiltration analysis (TIMER, ESTIMATE algorithm, CIBESORTE algorithm, among others), multi-omics data (proteomics, transcriptomics, immune-omics, single-cell RNA-seq, among others), and multiple ccRCC cohorts were used, bridging a molecular biology background and the clinical translation value. However, this study has several limitations. First, our study did not clarify the underlying mechanism of HK3 in the cellular glycolysis metabolism in ccRCC. Second, the enrolled patients should have been patients from a real-world cohort to more accurately validate the biomarker-predicting ICT responses.

## Conclusion

In conclusion, *HK3* activates the ccRCC microenvironment by stimulating the abundance of infiltrating monocytes/macrophages presenting surface markers, and may regulate the key molecular subgroups of immune checkpoint molecules of exhaustive T cells, prompting the microenvironmental characteristics of active anti-tumor immune responses. The large-scale data first revealed that *HK3* could predict the aggressive progression and a poor prognosis of ccRCC and improve the predictive outcomes of ccRCC patients receiving ICTs. These findings shed light on the tumor microenvironment and targeted therapies for ccRCC.

## Supplementary Material

Supplementary figures and table.

## Figures and Tables

**Table 1:** Clinicopathological characteristics baseline in relation to HK3 mRNA expression status in 910 ccRCC patients from TCGA and FUSCC cohorts

Characteristics, N (%)	TCGA cohort (N=533)	HK3 mRNA expression		χ^2^	*P*	FUSCC cohort (N=377)	HK3 mRNA expression		χ^2^	*P*
		High (N=230)	Low (N=303)				High (N=188)	Low (N=189)		
**Age**				1.635	0.201				0.643	0.423
≥60 years	245 (46.0)	160 (44.1)	85 (50.0)			125 (33.2)	122 (64.9)	130 (68.8)		
<60 years	288 (54.0)	203 (55.9)	85 (50.0)			252 (66.8)	66 (35.1)	59 (31.2)		
**Gender**				0.035	0.852				0.527	0.468
Male	345 (64.7)	234 (64.5)	111 (65.3)			250 (66.3)	128 (68.1)	122 (64.6)		
Female	188 (35.3)	129 (35.5)	59 (34.7)			127 (33.7)	60 (31.9)	67 (35.4)		
**pT stage**				7.278	**0.007**				31.22	**<0.001**
T1-T2	342 (64.2)	219 (60.3)	123 (72.4)			307 (81.4)	132 (70.2)	175 (92.6)		
T3-T4	191 (35.8)	144 (39.7)	47 (27.6)			70 (18.6)	56 (29.8)	14 (7.4)		
**pN stage**				1.041	0.308				12.117	**<0.001**
N0	240 (93.8)	166 (92.7)	74 (96.1)			331 (87.8)	154 (81.9)	177 (93.7)		
N1	16 (6.3)	13 (7.3)	3 (3.9)			46 (12.2)	34 (18.1)	12 (6.3)		
**pM stage**				4.239	**0.040**				39.493	**<0.001**
M0	448 (84.1)	297 (81.8)	151 (88.8)			308 (81.7)	130 (69.1)	178 (94.2)		
M1	85 (15.9)	66 (18.2)	19 (11.2)			69 (18.3)	58 (30.9)	11 (5.8)		
**AJCC stage †**				7,788	**0.005**				32.193	**<0.001**
I- II	324 (60.8)	206 (56.7)	118 (69.4)			291 (77.2)	122 (64.9)	169 (89.4)		
III-IV	209 (39.2)	157 (43.3)	52 (30.6)			86 (22.8)	66 (35.1)	20 (10.6)		
**ISUP grade**				19.765	**<0.001**				4.925	**0.026**
G1-G2	246 (46.6)	145 (40.1)	101 (60.8)			180 (47.7)	79 (42.0)	101 (53.4)		
G3-G4	282 (53.4)	217 (59.9)	65 (39.2)			197 (52.3)	109 (58.0)	88 (46.6)		

(TCGA, the Cancer Genome Atlas; FUSCC, Fudan University Shanghai Cancer Center; AJCC, the American Joint Committee on Cancer; TNM stage, Tumor size, Lymph Nodes affected, Metastases; P value less than 0.05 was marked in bold).

**Table 2:** Infiltrated levels of immune cell signatures with HK3 mRNA expression in ccRCC

Description	Gene markers	HK3 expression			
		None		Purity	
		Cor.	P	Cor.	P
CD8+ T cell	CD8A	0.382	****	0.335	****
	CD8B	0.382	****	0.34	****
T cell (general)	CD3D	**0.428**	****	0.386	****
	CD3E	**0.422**	****	0.373	****
	CD2	**0.420**	****	0.373	****
B cell	CD19	0.384	****	0.353	****
	CD79A	0.363	****	0.323	****
Monocyte	**CD86**	**0.551**	****	**0.518**	****
	**CD115 (CSF1R)**	**0.529**	****	**0.507**	****
TAM	CCL2	0.088	*	0.017	0.714
	**CD68**	**0.450**	****	**0.466**	****
	**IL10**	**0.511**	****	**0.486**	****
M1 Macrophage	INOS (NOS2)	-0.07	0.107	-0.119	*
	**IRF5**	**0.435**	****	**0.440**	****
	**CD80**	**0.593**	****	**0.593**	****
	COX2 (PTGS2)	0.061	0.158	0.017	0.721
M2 Macrophage	**CD163**	**0.51**	****	**0.512**	****
	**VSIG4**	**0.597**	****	**0.598**	****
	**MS4A4A**	**0.521**	****	**0.506**	****
Neutrophils	CD66b (CEACAM8)	0.101	*	0.121	**
	**CD11b (ITGAM)**	**0.627**	****	**0.604**	****
	CCR7	0.373	****	0.336	****
Natural killer cell	KIR2DL1	0.107	*	0.065	0.164
	KIR2DL3	0.103	*	0.086	0.065
	KIR2DL4	0.225	****	0.193	****
	KIR3DL1	0.059	0.172	0.056	0.23
	KIR3DL2	0.091	*	0.058	0.211
	KIR3DL3	0.112	**	0.098	*
	KIR2DS4	0.05	0.248	0.012	0.805
Dendritic cell	HLA-DPB1	**0.422**	****	0.397	****
	HLA-DQB1	0.267	****	0.228	****
	HLA-DRA	**0.422**	****	**0.400**	****
	HLA-DPA1	0.380	****	0.346	****
	BDCA-1 (CD1C)	0.212	****	0.156	***
	BDCA-4 (NRP1)	-0.098	*	-0.151	**
	**CD11c (ITGAX)**	**0.698**	****	**0.683**	****
Th1	T-bet (TBX21)	0.374	****	0.341	****
	STAT4	0.412	****	0.376	****
	STAT1	0.340	****	0.295	****
	IFN-γ (IFNG)	**0.420**	****	0.377	****
	TNF-α (TNF)	0.387	****	0.361	****
Th2	GATA3	0.122	**	0.095	*
	STAT6	0.196	****	0.233	****
	**STAT5A**	**0.438**	****	**0.418**	****
	IL13	0.205	****	0.197	****
Tfh	BCL6	0.175	****	0.191	****
	IL21	0.197	****	0.181	****
Th17	STAT3	0.084	0.052	0.063	0.177
	IL17A	0.066	0.126	0.03	0.521
Treg	**FOXP3**	**0.459**	****	**0.425**	****
	CCR8	0.386	****	0.357	****
	STAT5B	-0.09	*	-0.095	0.041
	TGFβ (TGFB1)	0.16	***	0.123	**
T cell exhaustion	**PD-1 (PDCD1)**	**0.483**	****	**0.455**	****
	**CTLA4**	**0.475**	****	**0.437**	****
	**LAG3**	**0.47**	****	**0.432**	****
	TIM-3 (HAVCR2)	0.190	****	0.152	**
	GZMB	0.377	****	0.320	****

TAM, tumor-associated macrophage; Th, T helper cell; Tfh, Follicular helper T cell; Treg, regulatory T cell; Cor, R value of Spearman's correlation; None, correlation without adjustment. Purity, correlation adjusted by purity; * P< 0.05; ** P< 0.01; *** P< 0.001; **** P< 0.0001; Cor. Value higher than 0.4 was considered as statistically significance and marked in bold.
