# Supplementary material for: Hexokinase 3 dysfunction promotes tumorigenesis and immune escape by upregulating monocyte/macrophage infiltration into the clear cell renal cell carcinoma microenvironment
Source: Int J Biol Sci. 2021 Jun 1;17(9):2205–22. doi: 10.7150/ijbs.58295 (PMC8241725; doi:10.7150/ijbs.58295)
Supplement: Supplementary file 1 — Supplementary figures and table. [file ijbsv17p2205s1.pdf]

Supplementary Figure 1. Univariate Cox analysis suggested pathological TNM stage, AJCC stages, ISUP grade and HK3 expression as prognostic indicators in 533 ccRCC patients from TCGA cohort.

Supplementary Figure 2. *HK3* plays an important role in the immune microenvironment of various cancers.

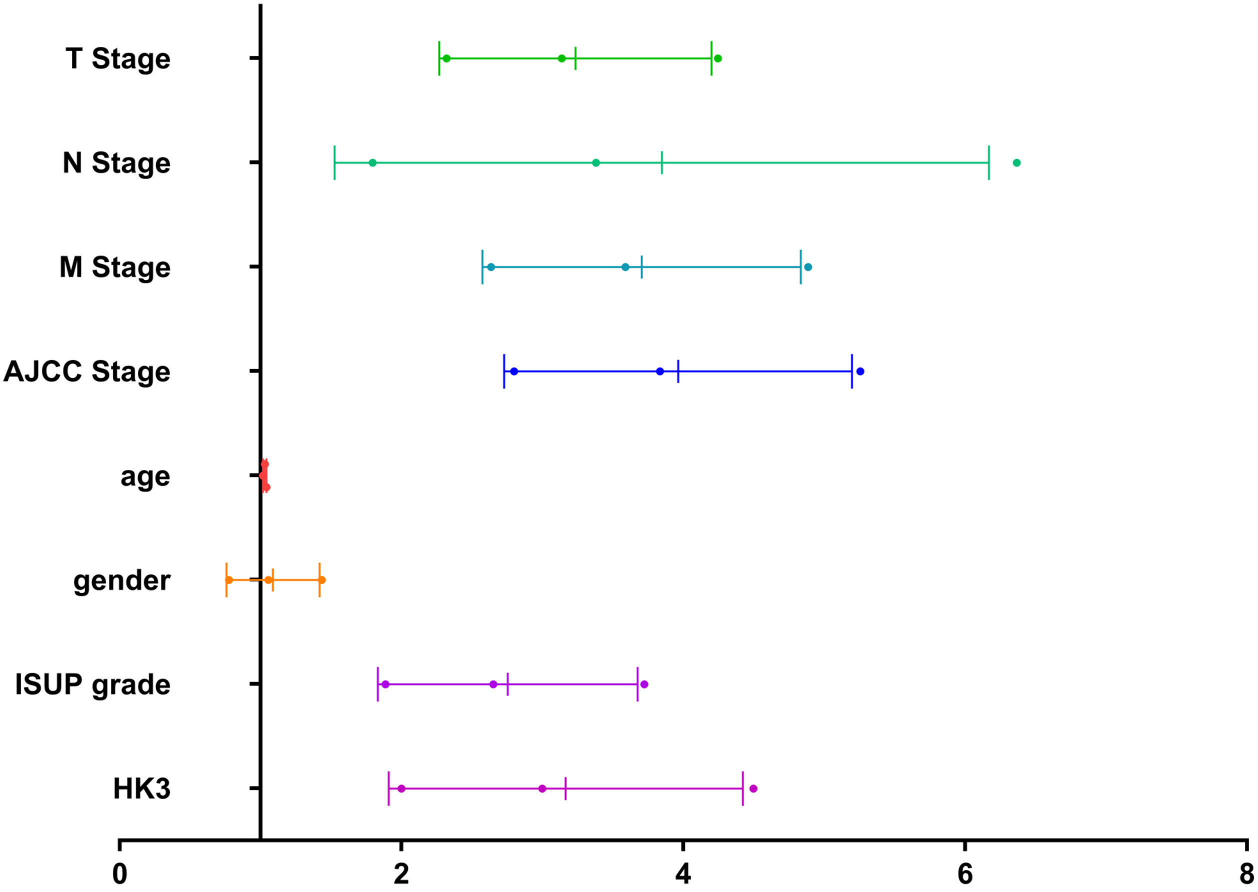
T Stage
N Stage
M Stage
AJCC Stage
age
gender
ISUP grade
HK3
0
2
4
6
8

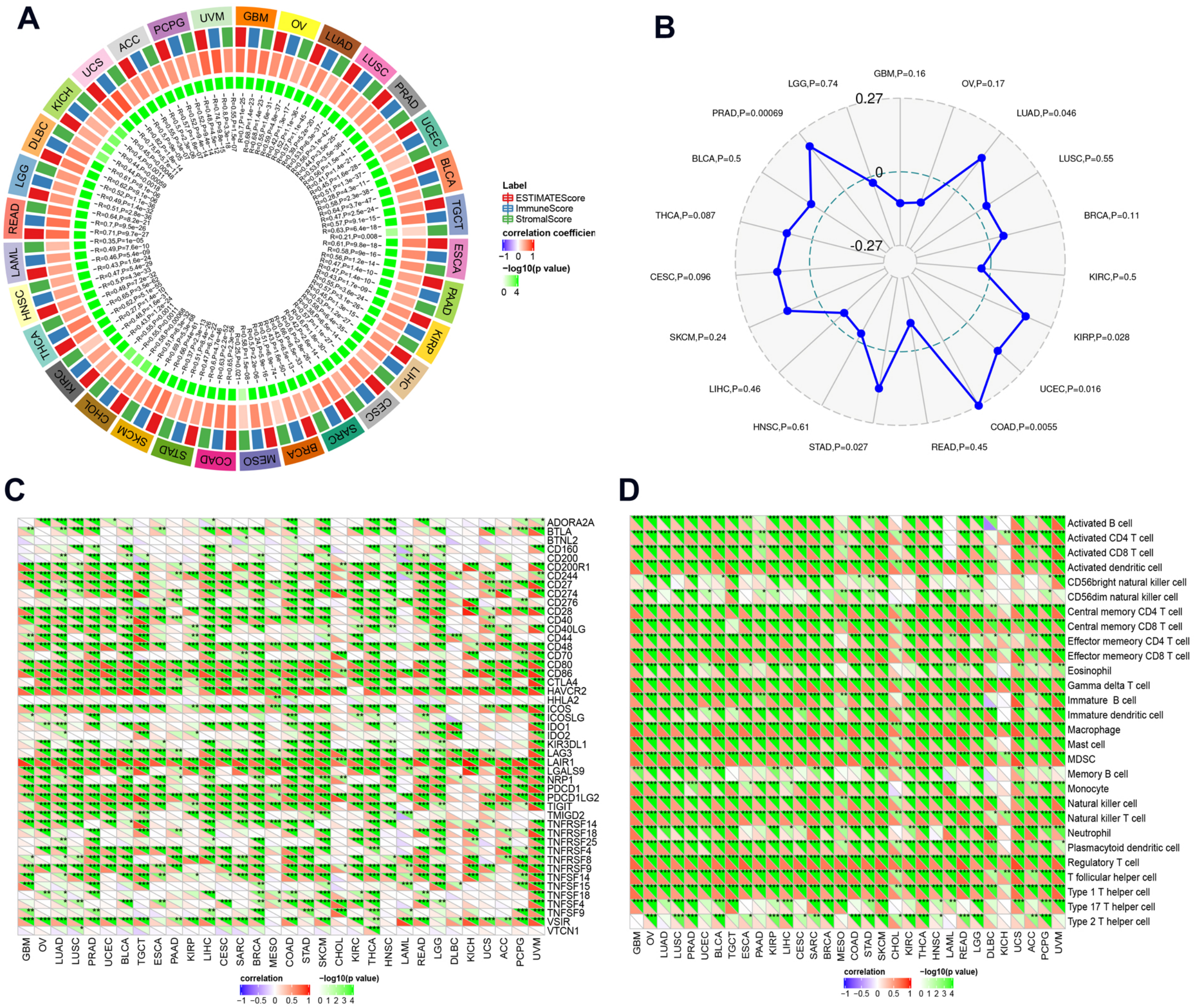

A
Label
ESTIMATEScore
ImmuneScore
StromalScore
correlation coefficient
-1 0 1
-log10(p value)
0 4
GBM OV LUAD LUSC PRAD UCEC BLCA TGCT ESCA PAAD KIRP LIHC CESC SARC BRCA MESO COAD STAD SKCM CHOL KIRC THCA HNSC LAML READ LGG DLBC KICH UCS ACC PCPG UVM
B
GBM,P=0.16
OV,P=0.17
LUAD,P=0.046
LUSC,P=0.55
BRCA,P=0.11
KIRC,P=0.5
KIRP,P=0.028
UCEC,P=0.016
COAD,P=0.0055
READ,P=0.45
STAD,P=0.027
HNSC,P=0.61
LIHC,P=0.46
SKCM,P=0.24
CESC,P=0.096
THCA,P=0.087
BLCA,P=0.5
PRAD,P=0.00069
LGG,P=0.74
0.27
0
-0.27
C
ADORA2A
BTLA
BTNL2
CD160
CD200
CD200R1
CD244
CD27
CD274
CD276
CD28
CD40
CD40LG
CD44
CD48
CD70
CD80
CD86
CTLA4
HAVCR2
HHLA2
ICOS
ICOSLG
IDO1
IDO2
KIR3DL1
LAG3
LAIR1
LGALS9
NRP1
PDCD1
PDCD1LG2
TIGIT
TMIGD2
TNFRSF14
TNFRSF18
TNFRSF25
TNFRSF4
TNFRSF8
TNFRSF9
TNFSF14
TNFSF15
TNFSF18
TNFSF4
TNFSF9
VSIR
VTCN1
GBM OV LUAD LUSC PRAD UCEC BLCA TGCT ESCA PAAD KIRP LIHC CESC SARC BRCA MESO COAD STAD SKCM CHOL KIRC THCA HNSC LAML READ LGG DLBC KICH UCS ACC PCPG UVM
correlation
-1 -0.5 0 0.5 1
-log10(p value)
0 1 2 3 4
D
Activated B cell
Activated CD4 T cell
Activated CD8 T cell
Activated dendritic cell
CD56bright natural killer cell
CD56dim natural killer cell
Central memory CD4 T cell
Central memory CD8 T cell
Effector memeory CD4 T cell
Effector memeory CD8 T cell
Eosinophil
Gamma delta T cell
Immature B cell
Immature dendritic cell
Macrophage
Mast cell
MDSC
Memory B cell
Monocyte
Natural killer cell
Natural killer T cell
Neutrophil
Plasmacytoid dendritic cell
Regulatory T cell
T follicular helper cell
Type 1 T helper cell
Type 17 T helper cell
Type 2 T helper cell
GBM OV LUAD LUSC PRAD UCEC BLCA TGCT ESCA PAAD KIRP LIHC CESC SARC BRCA MESO COAD STAD SKCM CHOL KIRC THCA HNSC LAML READ LGG DLBC KICH UCS ACC PCPG UVM
correlation
-1 -0.5 0 0.5 1
-log10(p value)
0 1 2 3 4

Supplementary Table 1. Univariate Cox logistic regression analysis of PFS and OS in 377 ccRCC patients from FUSCC cohort (PFS: progression-free survival; OS: overall survival; ccRCC: clear cell renal cell carcinoma; FUSCC: Fudan University Shanghai Cancer Center)

Covariates	PFS			OS		
	HR	95% CI	*P* value	HR	95% CI	*P* value
Age	1.013	1.001-1.025	**0.029**	1.015	1.002-1.028	**0.027**
Lateral	1.029	0.783-1.352	0.838	0.981	0.719-1.339	0.903
BMI	0.905	0.813-1.051	0.091	0.947	0.886-1.011	0.076
pT stage (ref. T1-T2)	6.555	4.790-8.970	**<0.001**	7.968	5.689-11.160	**<0.001**
pN stage (ref. N0)	7.810	5.444-11.204	**<0.001**	8.929	6.052-13.174	**<0.001**
pM stage (ref. M0)	7.864	5.732-10.790	**<0.001**	10.197	7.204-14.433	**<0.001**
AJCC stage (ref. I-II)	9.670	7.027-13.307	**<0.001**	12.994	9.066-18.624	**<0.001**
ISUP grade (ref. 1-2)	2.742	2.049-3.670	**<0.001**	3.370	2.372-4.788	**<0.001**
HK3 expression (ref. Low)	2.852	2.131-3.817	**<0.001**	2.999	1.999-4.497	**<0.001**
